# Expression of EZH2 and Ki-67 in colorectal cancer and associations with treatment response and prognosis

**DOI:** 10.1038/sj.bjc.6605333

**Published:** 2009-09-22

**Authors:** Ø Fluge, K Gravdal, E Carlsen, B Vonen, K Kjellevold, S Refsum, R Lilleng, T J Eide, T B Halvorsen, K M Tveit, A P Otte, L A Akslen, O Dahl

**Affiliations:** 1 Department of Oncology, Haukeland University Hospital, Bergen, Norway; 2 Department of Pathology, Haukeland University Hospital, Bergen, Norway; 3 Department of Surgery, Ullevål University Hospital, Oslo, Norway; 4 Department of Surgery, University of Northern Norway, Tromsø, Norway; 5 Department of Pathology, Stavanger University Hospital, Stavanger, Norway; 6 Laboratory for Pathology AS, Oslo, Norway; 7 Department of Pathology, Buskerud Central Hospital, Drammen, Norway; 8 Department of Pathology, The National Hospital, Oslo, Norway; 9 Department of Pathology, St Olav's Hospital, Trondheim, Norway; 10 Department of Oncology, Ullevål University Hospital, Oslo, Norway; 11 Department of Biochemistry, Swammerdam Institute for Life Sciences, University of Amsterdam, Amsterdam, The Netherlands; 12 The Gade Institute, Department of Pathology, University of Bergen, Bergen, Norway; 13 Section of Oncology, Institute of Medicine, University of Bergen, Bergen, Norway

**Keywords:** colorectal cancer, adjuvant chemotherapy, prediction of outcome, EZH2, ki-76, immunehistochemistry

## Abstract

**Background:**

Enhancer of zeste homologue 2 (EZH2) is a member of the Polycomb group of genes that is involved in epigenetic silencing and cell cycle regulation.

**Methods:**

We studied EZH2 expression in 409 patients with colorectal cancer stages II and III. The patients were included in a randomised study, and treated with surgery alone or surgery followed by adjuvant chemotherapy.

**Results:**

EZH2 expression was significantly related to increased tumour cell proliferation, as assessed by Ki-67 expression. In colon cancer, strong EZH2 expression (*P*=0.041) and high proliferation (⩾40%; *P*=0.001) were both associated with better relapse-free survival (RFS). In contrast, no such associations were found among rectal cancers. High Ki-67 staining was associated with improved RFS in colon cancer patients who received adjuvant chemotherapy (*P*=0.001), but not among those who were treated by surgery alone (*P*=0.087). In colon cancers stage III, a significant association between RFS and randomisation group was found in patients with high proliferation (*P*=0.046), but not in patients with low proliferation (*P*=0.26). Multivariate analyses of colon cancers showed that stage III (hazard ratio (HR) 4.00) and high histological grade (HR 1.80) were independent predictors of reduced RFS, whereas high proliferation indicated improved RFS (HR 0.55).

**Conclusion:**

Strong EZH2 expression and high proliferation are associated features and both indicate improved RFS in colon cancer, but not so in rectal cancer.

Colorectal cancer is among the most common malignancies, and is one of the leading causes of cancer deaths in the western world. Although adequate surgery is the main treatment, adjuvant chemotherapy has since 1990 been considered standard to patients with lymph node metastases (Wolmark *et al*, 1988; Laurie *et al*, 1989; Moertel *et al*, 1990; Andre *et al*, 2004; de Gramont *et al*, 2007). The search for molecular markers with prognostic or especially predictive effect is important to select patients for proper therapy, and to avoid unnecessary treatment with toxic side effects.

Enhancer of zeste homologue 2 (EZH2) is a member of the Polycomb group of genes (Chen *et al*, 1996), encoding a transcriptional repressor as part of the histone methyltransferase complex (van der Vlag and Otte, 1999), with important functions in epigenetic silencing and cell cycle regulation. EZH2 is an E2F-regulated gene (Bracken *et al*, 2003), and activated p53 may downregulate EZH2 transcription through repression of the promoter (Tang *et al*, 2004). Overexpression of EZH2 protein has been shown in several carcinomas, and has been associated with increased tumour cell proliferation and worse outcome in breast cancer (Collett *et al*, 2006), melanomas, endometrial carcinomas (Bachmann *et al*, 2006), and also in hormone-refractory prostate cancer (Bryant *et al*, 2007).

In this study we analysed the expression of EZH2 protein and the proliferation marker Ki-67, using tissue microarrays and immunohistochemistry, in 409 patients with colorectal cancer in stages II and III, randomised to either surgery alone or to surgery and adjuvant chemotherapy with 5-fluorouracil (5-FU) and levamisol (FLev).

## Materials and methods

From December 1992 to October 1996, a total of 425 patients with colorectal cancer were included in a prospective randomised clinical study. The inclusion criteria and patient characteristics have been presented earlier (Dahl *et al*, 2009). In brief, before inclusion the patients should have a histopathologically confirmed radical surgical resection of the primary tumour and regional lymph nodes. The patients should have no evidence of distant metastases evaluated by chest X-ray, ultrasound or computed tomography of the abdomen, and blood tests including carcinoembryonal antigen (CEA). The patients were between 18 and 75 years of age, and had no concurrent diseases precluding the administration of systemic chemotherapy. Patients with primary tumour localisation in the colon or the rectum, and histopathological tumour–node–metastasis (TNM) stage II (penetration of muscularis propria) or TNM stage III (with lymph node metastases) were eligible for inclusion. The patients were randomly allocated to the treatment groups, and were treated according to this schedule. None of the patients with rectal cancer received preoperative radiotherapy. The inclusion criteria were adequately met for 412 patients, and these were included in the statistical analyses. All data were collected at the Regional Research Office and then centralised to the office in Bergen, Norway, where all the data handling took place. All patients had to give their written informed consent before randomisation. The study was approved by the regional ethics committee, The Norwegian Medicines Control Authority, and the Data Inspectorate.

### Chemotherapy and follow-up

A loading course of 5-FU was started within 42 days from the date of surgery, giving 450 mg m^−2^ intravenously (i.v.) daily for 5 days and levamisol 50 mg × 3 for the first 3 days. After 3 weeks, maintenance therapy was started with weekly i.v. 5-FU 450 mg m^−2^, combined with levamisol 50 mg × 3 for 3 days every second week, for a total maintenance therapy period of 48 weeks. The patients were thereafter observed regularly every 6 months with clinical examination including chest X-ray, ultrasound of the abdomen, and relevant endoscopical examination, including coloscopy every 3 years. The follow-up time was scheduled for 5 years. For 272 patients without relapse, the median follow-up time was 7 years.

### Tissue microarray and immunohistochemistry

The paraffin-embedded and formalin-fixed tissue specimens were available from 409 out of 412 eligible patients for tissue microarrays (TMA). Cylinders, 1 mm in diameter, were punched from the representative areas of tumour cell infiltration in the primary tumours, from macroscopically normal colon or rectal mucosa, and from lymph nodes with synchronous metastases, which were mounted in recipient paraffin blocks using a standard precision instrument (Manual Tissue Arrayer MTA-1, Beecher Instruments, Inc., Sun Prairia, WI, USA). A total of about 1400 cylinders were mounted, varying from one to three representative samples from each tumour, and one from normal colon or rectal mucosa, and from lymph node metastases.

EZH2 staining was performed on a DAKO autostainer using the EnVision method, and the TMA slides were incubated for 1 h using the monoclonal antibody (clone M18) specific for EZH2, as described (Collett *et al*, 2006). Ki-67 immunostaining was performed on formalin-fixed and paraffin- embedded tissue using 5μm sections. After microwave antigen retrieval (boiling for 15 min at 400 W) in Tris-ethylenediaminetetraacetic acid buffer (pH 9.0), the slides were incubated for 30 min at room temperature with the monoclonal mouse anti-human Ki-67 antibody (clone MIB-1) Cat. no. M 7240 (Dako, Copenhagen) diluted 1:250, and stained with horseradish peroxidase (HRP) EnVision (Dako) for 30 min at room temperature. HRP was localised using the diaminobenzidine tetrachloride peroxidase reaction and the sections were counterstained with Mayer's haematoxylin.

For EZH2, tumour cell nuclear staining intensity (score 0–3), and the fraction of positive tumour cells (score 0–3), were identified by a skilled pathologist blinded from the follow-up data. The EZH2 fraction of positive tumour cells was categorised as follows: 0, 0%; 1, <10%; 2, 10–50%; and 3, >50%. The EZH2 staining intensity was recorded as: 0, no staining; 1, weak; 2, moderate; and 3, strong staining. A staining index (SI) was generated from the product (0–9), and then categorised in either three (SI 0–1, 2–4, 6–9) or two (SI 0–3, 4–9) groups. For Ki-67 immunohistochemistry, the percentage of positive tumour cells was assessed in several representative visual fields of each tumour. A cutoff was then chosen at the median value, dividing the samples in low (<40% positive tumour cells) or high nuclear Ki-67 expression (⩾40%). This cutoff is also supported by another study (Salminen *et al*, 2005).

### Statistics

The survival data were calculated according to the product-limit procedure (Kaplan–Meier method), and *P*-values were based on the log-rank test, with a *P*-value of <0.05 considered statistically significant in two-tailed tests. Kaplan–Meier analyses for survival were performed for relapse-free survival (RFS) and for cancer-specific survival (CSS), including death for colorectal cancer and death from treatment-related complications as outcome events.

The associations between EZH2 protein expression and different categorical clinicopathological variables were analysed by Pearson's chi-square test. Cox’ regression analysis was performed to assess independent prognostic factors for RFS in colon cancer, including the variables stage (II *vs* III), Ki-67 staining (<40% *vs* ⩾40% positive tumour cell nuclei), tumour differentiation (high or moderate differentiation *vs* poor differentiation), randomisation group (adjuvant chemotherapy *vs* surgery alone), and tumour localisation (proximal *vs* distal colon), and the variables were included in a single step. The data handling was performed by the SPSS software package (version 16.0).

## Results

Among the 412 patients included, 290 had colon cancer (185 in stage II and 105 in stage III), and 122 had rectal cancer (62 in stage II and 60 in stage III). In all, 206 patients were randomised to each group (with or without adjuvant chemotherapy), 215 were men and 197 women, and the mean age at randomisation was 61.3 years (range 28.4–75.1). The details of the patient characteristics have been described earlier (Dahl *et al*, 2009).

For all patients included, that is, both colon and rectal cancer (stages II and III), Kaplan–Meier analyses showed significantly better RFS for tumour localisation in colon *vs* rectum (5-year RFS 71 *vs* 55%, *P*=0.003), for tumour grade (5-year RFS 67% for high or moderate grade *vs* 55% for poorly differentiated, *P*=0.022), for stage (5-year RFS 82% for stage II *vs* 42% for stage III, *P*<0.001), for EZH2 expression (5-year RFS 79% for index 4–9 *vs* 63% for index 0–3, *P*=0.038), and for Ki-67 expression (5-year RFS 73% for ⩾40% *vs* 59% for <40%, *P*=0.003). In the complete patient series, there were no significant associations between RFS and the randomisation group (5-year RFS 69% for adjuvant treatment *vs* 63% for surgery alone, *P*=0.24), or between RFS and age or sex.

Within patients with colon cancer (stages II and III), the univariate associations between clinicopathological variables and RFS are shown in Table 1. As expected, the presence of lymph node metastases (stage III) was associated with a reduced 5-year RFS rate (*P*<0.001). Adjuvant therapy was significantly associated with better RFS in colon cancer stage III (*P*=0.012), but not when analysing colon cancer stages II and III together (*P*=0.35).

**Figure 1:**
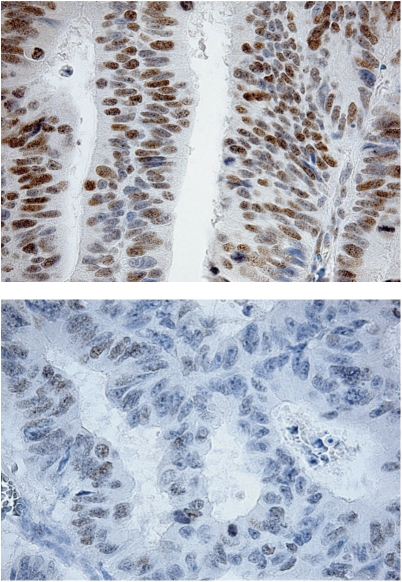
Strong (top) and weak (bottom) nuclear EZH2 staining in tumour cells.

**Figure 2:**
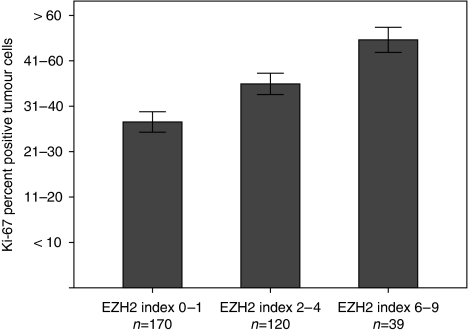
Association between EZH2 staining index and the percentage of Ki-67-positive tumour cells.

EZH2 protein expression is shown in Figure 1. The associations of EZH2 protein expression and the clinicopathological variables, for patients with colon cancer stages II and III, are shown in Table 2. There was a significant association between a high EZH2 index and increased tumour cell proliferation, as assessed by Ki-67 expression (*P*<0.001; Figure 2). There were no significant associations between EZH2 index and the sex, age, tumour localisation (proximal *vs* distal colon), histological grade, randomisation group, or tumour stage. The proportion of patients with stage III was 41.4% in those with a low EZH2 index (0–1) as compared with 34.5% in those with index 2–4, and 27.6% in patients with a high index, but this was not significant (*P* for trend 0.14).

**Figure 3:**
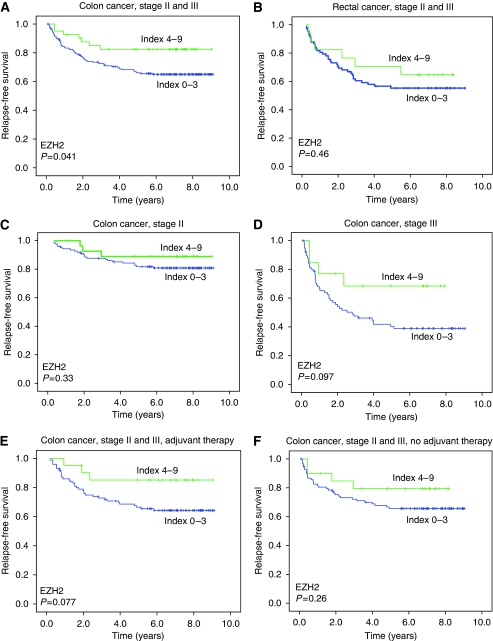
Kaplan–Meier survival analyses for high EZH2 staining index (4–9) *vs* low EZH2 index (0–3) in colon cancer stages II and III (**A**), rectal cancer stages II and III (**B**), colon cancer stage II (**C**), colon cancer stage III (**D**), in colon cancer patients of stages II and III receiving adjuvant chemotherapy (**E**), and in colon cancer patients of stages II and III with no adjuvant therapy (**F**). *P*-values are from log-rank tests.

Figure 3 shows Kaplan–Meier curves for the effect of EZH2 expression (high index 4–9, *vs* low index 0–3). In colon cancer stages II and III, a high EZH2 index was significantly associated with a better RFS (Figure 3, panel A, *P*=0.041 by the log-rank test), and with a trend for better CSS (*P*=0.069). In rectal cancer stages II and III, no differences in RFS or CSS were detected for high *vs* low EZH2 index (Figure 3, panel B, *P*=0.46). A corresponding subgroup analysis within colon cancer stage III patients showed a trend for improved RFS with high EZH2 index (*P*=0.097), whereas a similar analysis for CSS was not significant (*P*=0.14; Figure 3, panels C and D). In colon cancer stages II and III, there were trends for better RFS (*P*=0.077) and CSS (*P*=0.085) with a high EZH2 index in patients receiving adjuvant chemotherapy (Figure 3, panel E), but not in patients with no adjuvant therapy (Figure 3, panel F).

**Figure 4:**
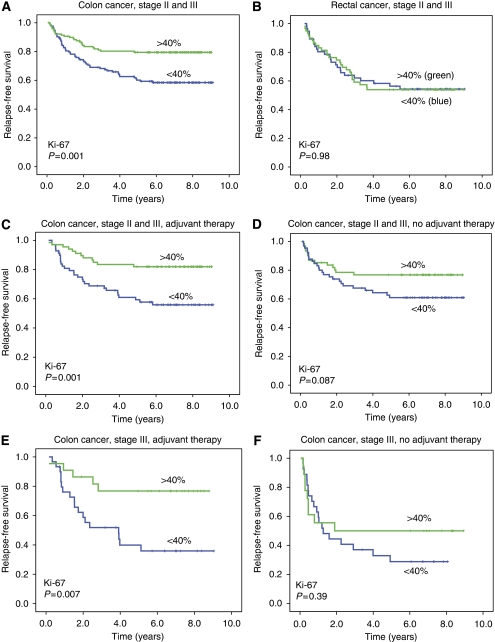
Kaplan–Meier survival analyses for high Ki-67 expression (⩾40% positive tumour cells) *vs* low Ki-67 expression (<40% tumour cells) in colon cancer stages II and III (**A**), rectal cancer stages II and III (**B**), colon cancer stages II and III receiving adjuvant therapy (**C**), colon cancer stages II and III with no adjuvant therapy (**D**), colon cancer stage III receiving adjuvant therapy (**E**), and colon cancer stage III with no adjuvant therapy (**F**). *P*-values are from log-rank tests.

Ki-67 expression in >40% of the tumour cells was significantly associated with improved RFS (*P*=0.001) and CSS (*P*=0.001) in patients with colon cancer stages II and III (Figure 4, panel A), but not in rectal cancer (Figure 4, panel B, *P*=0.98 for RFS, *P*=0.96 for CSS). In Figure 4, panels C and D, subgroup analyses in patients receiving adjuvant treatment and those randomised to surgery alone (stages II and III) are shown. A high Ki-67 expression was significantly associated with an improved RFS (*P*=0.001, Figure 4, panel C) and improved CSS (*P*=0.003) in the adjuvant group, but only with a trend for better RFS (*P*=0.087) and with no significant affect on CSS (*P*=0.11) in the group treated with surgery alone (Figure 4, panel D). Correspondingly, in patients with colon cancer stage III, Kaplan–Meier curves showed significant associations between a high Ki-67 (⩾40%) and better RFS (*P*=0.007) and CSS (*P*=0.017) in the group that was given adjuvant treatment, but not in the group without adjuvant chemotherapy. In the adjuvant group, 5-year RFS was 77% in those with a high Ki-67, and 36% in those with low Ki-67 (*P*=0.007; Figure 4, panel E). The corresponding figures for 5-year CSS were 81% in patients with a high Ki-67, and 51% in those with low Ki-67 (*P*=0.017). In the group without adjuvant therapy, 5-year RFS was 50% in those with a high Ki-67 *vs* 29% in those with a low Ki-67 (*P*=0.39; Figure 4, panel F), whereas the figures for CSS were 55 and 44% (*P*=0.38).

The results of Cox’ multivariate analyses, including 256 out of 284 patients with colon cancer stages II and III, are shown in Table 3. The significant independent predictors for reduced RFS were stage III (hazard ratio (HR) 4.00, 95% confidence interval (CI) 2.50–6.39), high histological tumour grade (HR 1.80, 95% CI 1.09–2.98), whereas a high Ki-67 expression (⩾40%) was independently associated with an improved RFS (HR 0.55, 95% CI 0.34–0.89). Randomisation group (*P*=0.31), and tumour localisation (proximal *vs* distal colon, *P*=0.27) were not independently associated with RFS in colon cancer stages II and III.

**Figure 5:**
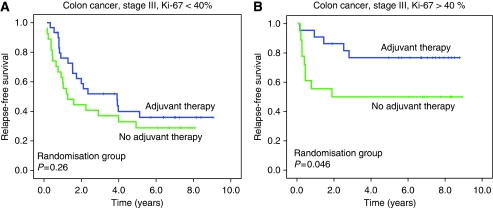
Kaplan–Meier survival analyses for colon cancer stage III patients receiving adjuvant therapy *vs* colon cancer stage III patients with no adjuvant therapy. Separate panels for patients with low Ki-67 expression (<40% positive tumour cells) (**A**), and for patients with high Ki-67 expression (⩾40% positive tumour cells) (**B**). *P*-values are from log-rank tests.

Finally, in Figure 5 are presented the results of Kaplan–Meier analyses in 98 patients with colon cancer stage III, with separate panels for those with a high Ki-67 expression (⩾40%, *n*=41) and those with a low Ki-67 expression (<40%, *n*=57), showing the effect of randomisation group on RFS. In patients with a low Ki-67 protein expression in the primary tumours, no association was found between randomisation group and RFS (Figure 5, panel A, *P*=0.26). In contrast, in the group of patients with colon cancer stage III and a high Ki-67 expression, 5-year RFS in those receiving adjuvant chemotherapy was 77% (s.e. 0.09) as compared with 50% (s.e. 0.12) in those treated with surgery without adjuvant chemotherapy (*P*=0.046). The corresponding figures for 5-year CSS were 81% in patients receiving adjuvant chemotherapy (s.e. 0.09) compared with 56% (s.e. 0.12) in those with surgery alone (*P*=0.070).

## Discussion

In this study, patients with colorectal cancer stages II and III were randomised to either surgery alone, or the addition of 1 year of adjuvant therapy with FLev. This chemotherapy regimen is no longer a standard practice for adjuvant treatment of colon cancer stage III, because of the introduction of more effective regimens, including the cytotoxic drug oxaliplatin (Andre *et al*, 2004). However, a strength of this study is that adjuvant FLV chemotherapy was compared with surgery alone, which would be considered unethical nowadays.

We studied the affect of EZH2 protein expression and the proliferation marker Ki-67 on survival. EZH2 expression was significantly correlated with tumour cell proliferation as assessed by the percentage of Ki-67-positive cells, but not to other clinicopathological variables. EZH2 is a cell cycle regulator necessary for G2-M transition and is an E2F-regulated gene (Bracken *et al*, 2003). The association between proliferation and EZH2 expression has been shown in other malignancies such as breast cancer and melanomas (Bachmann *et al*, 2006; Collett *et al*, 2006). In these malignancies, EZH2 expression in tumour cells was linked to an aggressive clinical behaviour and worse prognosis. In addition, in prostate cancer cells, EZH2 was found to promote invasiveness and proliferation (Bryant *et al*, 2007). In our study, a high EZH2 index was associated with a better RFS in colon cancer stages II and III, but not in rectal cancer. Subgroup analyses showed that this benefit was primarily in patients with colon cancer stage III, but this did not reach statistical significance.

Unexpectedly, and in line with our EZH2 data, Ki-67 protein expression in >40% of the tumour cells was also associated with a better RFS in colon cancer stages II and III. This association was not observed in rectal cancer patients. Another study of rectal cancers has shown significant association between high Ki-67 (>40%) and improved survival (Salminen *et al*, 2005). The prognostic value of Ki-67 was significant in colon cancer patients with stage III who were receiving adjuvant chemotherapy. However, in colon cancer patients treated with surgery without adjuvant chemotherapy, only a trend for improved RFS was found for those with a high Ki-67 expression. Thus, a high tumour cell proliferation rate, as assessed by Ki-67 immunohistochemistry, is not an adverse prognostic marker in colorectal cancer stages II and III patients receiving adjuvant treatment. In fact, for colon cancer stages II and III, a high Ki-67 expression is associated with an improved RFS.

Among the colon cancer patients, the percentage of stage III patients was higher in those with a low Ki-67 expression than in those with a high Ki-67 (42.5% *vs* 31.8%), but this difference was not significant (*P*=0.07). In addition, the multivariate survival analyses, performed on colon cancer patients stages II and III, showed that a high Ki-67 immunostaining was an independent prognostic variable for an improved RFS and TNM stage and histological tumour grade.

Interestingly, the survival analyses performed separately for colon cancer stage III patients, and stratified according to Ki-67 expression, showed a significant association between RFS and the randomisation group only for patients with a high Ki-67 expression. Thus, in colon cancer stage III patients, a high Ki-67 expression (>40%) seems to be a predictive marker for the effect of 12-month adjuvant chemotherapy with FLV. EZH2 protein expression was not a significant predictive marker in this aspect.

The prognostic value of Ki-67 in different malignancies has been reviewed (Brown and Gatter, 2002). The most consistent data for an adverse prognostic value of a high Ki-67 have been reported for breast cancer (Baak *et al*, 2009), lung cancer, and sarcomas. For cervical cancer and prostate cancer, the reports on the associations between clinical outcome and Ki-67 expression have been varying (Brown and Gatter, 2002).

In colorectal cancer, data have, to some extent, been contradictory. Several studies have reported no prognostic value of Ki-67 expression. One study reported an association between a low tumour cell proliferation rate at the invasive margin and poor prognosis in colorectal cancer TNM stage II (Palmqvist *et al*, 1999), whereas others reported an adverse prognostic value of a high Ki-67 after curative resection for colorectal cancer (Kimura *et al*, 2000). In accordance with our data, a study of prognostic markers in colon cancer stage II and III, treated with surgery with or without adjuvant 5-FU and leucovorin (calcium folinate) therapy, showed an improved outcome in patients with a high percentage of Ki-67-positive tumour cells (Allegra *et al*, 2003). As a possible explanation, more rapidly proliferating tumour cells may be more vulnerable to chemotherapy-induced tumour cell death.

We conclude that increased EZH2 protein expression and Ki-67 expression in >40% of the tumour cells are both associated with an improved RFS in colon cancer stages II and III, but not so in rectal cancer. In addition, in colon cancer stage III, a high Ki-67 immunostaining in >40% of the tumour cells seems to be a predictive marker to the effect of adjuvant chemotherapy using FLV.

## Figures and Tables

**Table 1:** Univariate associations of variables with relapse-free survival within colon cancer, TNM stages II and III

	**No.**	**Relapse**	**5-year relapse- free survival (%)**	**s.e. (%)**	***P*-value**
*Age*					NS
⩽60 years	113	33	71	4	
>60 years	177	52	71	4	
					
*Sex*					NS
Women	152	48	69	4	
Men	138	37	73	4	
					
*TNM stage*					<0.001
Stage II	185	31	83	3	
Stage III	105	54	48	5	
					
*Randomisation group* a					NS
Adjuvant therapy	149	41	73	4	
Surgery alone	141	44	68	4	
					
*Randomisation group* b					0.012
Adjuvant therapy	54	23	58	7	
Surgery alone	51	31	37	7	
					
*Ki-67*					0.001
⩽40%	134	54	60	4	
>40%	129	26	80	4	
					
*EZH2*					0.041
Index 0–3	200	68	66	3	
Index 4–9	41	7	82	6	
					
*Histological type*					NS
Adenocarcinoma	250	74	70	3	
Variant^c^	32	6	80	7	
					
*Histological grade*					0.025
High/moderate	228	62	73	3	
Poor differentiation	56	22	59	7	
					
*Tumour localisation*					NS
Proximal colon	161	41	74	4	
Distal colon	129	44	67	4	

Abbreviations: EZH2*=*enhancer of zeste homologue 2; NS=not significant; TNM=tumor–node–metastasis.

a Including both TNM stages II and III.

b Including TNM stage III.

c Variant histology includes mucinous and signet-ring carcinomas.

**Table 2:** The proportion of cases (%) for different clinical and histological variables, according to EZH2 protein expression (A) and Ki-67 protein expression (B)

**(A)**				
	**EZH2 protein expression**			
**Variable**	**Index 0–1, *n*=124**	**Index 2–4, *n*=87**	**Index 6–9, *n*=29**	*P*-value ^*^
Sex (*n*=240, female, %)	55.6	54.0	44.8	NS
Age (*n*=240, mean, years)	62.2	61.6	63.2	NS
Tumour localisation *(n*=240, proximal colon, %)	52.4	58.6	55.2	NS
Tumour stage *(n*=240, T3 and T4, %)	93.6	97.4	93.1	NS
Histological type^a^ (*n*=232, adenocarcinoma, %)	93.4	81.9	92.9	0.028
Histological grade (*n*=235, poorly differentiated, %)	18.7	19.0	17.9	NS
TNM stage (*n*=240, stage III, %)	41.4	34.5	27.6	0.14^**^
Ki-67 (*n*=230, >40% tumour cells positive, %)	34.7	56.6	96.6	<0.001
Randomisation group (*n*=240, adjuvant chemotherapy, %)	47.6	55.2	51.7	NS
				
**(B)**				
	**Ki-67 protein expression**			
**Variable**	**<40%, *n*=134**	**⩾40 %, *n*=129**	P-value ^*^	
Sex (*n*=263, female, %)	52.2	54.3	NS	
Age (*n*=263, mean, years)	61.6	61.6	NS	
Tumour localisation (*n*=263, proximal colon, %)	56.0	56.6	NS	
Tumour stage (*n*=263, T3 and T4, %)	93.3	96.9	NS	
Histological type^a^ (*n*=256, adenocarcinoma, %)	91.6	87.2	NS	
Histological grade (*n*=257, poorly differentiated, %)	21.1	16.9	NS	
TNM stage (*n*=263, stage III, %)	42.5	31.8	0.07	
EZH2 protein expression (=230, index 4–9, %)	6.1	29.1	<0.0001	
Randomisation group (*n*=263, adjuvant chemotherapy, %)	51.5	51.9	NS	

Abbreviations: EZH2*=*enhancer of zeste homologue 2; NS=not significant; TNM=tumour–node–metastasis.

Analyses include patients with colon cancer in TNM stages II and III.

^*^P-values from chi-square statistics. NS: *P*>0.10.

^**^P for trend.

a Other histological types include mucinous adenocarcinoma and signet-ring cell carcinoma.

**Table 3:** Independent predictors for relapse-free survival in 256 patients with colon cancer TNM stages II and III, according to Cox regression model

**Variable**	**Hazard ratio (95% CI)**	***P*-value**
*TNM stage*		<0.0001
Stage II	1	
Stage III	4.00 (2.50–6.39)	
		
*Ki-67 staining*		0.014
<40%	1	
⩾40%	0.55 (0.34–0.89)	
		
*Tumour grade*		0.022
High or moderate differentiation	1	
Poor differentiation	1.80 (1.09–2.98)	
		
*Randomisation group*		0.31
No adjuvant therapy	1	
Adjuvant chemotherapy	0.80 (0.51–1.24)	
		
*Tumour localisation*		0.27
Proximal colon	1	
Distal colon	1.28 (0.82–1.99)	

Abbreviations: CI=confidence interval; TNM=tumor–node–metastasis.

Cox analysis (enter) was based on 256 out of 284 patients with colon cancer TNM stages II and III, and 28 cases had missing data for one variable.
